# Review of the role of additional treatments including oseltamivir, oral steroids, macrolides, and vitamin supplementation for children with severe pneumonia in low- and middle-income countries

**DOI:** 10.7189/jogh.12.10005

**Published:** 2022-08-22

**Authors:** Maeve Hume-Nixon, Hamish Graham, Fiona Russell, Kim Mulholland, Amanda Gwee, ARI Review group, Trevor Duke, Hamish Graham, Steve Graham, Amy Gray, Amanda Gwee, Claire von Mollendorf, Kim Mulholland, Fiona Russell, Maeve Hume-Nixon, Saniya Kazi, Priya Kevat, Eleanor Neal, Cattram Nguyen, Alicia Quach, Rita Reyburn, Kathleen Ryan, Patrick Walker, Chris Wilkes, Poh Chua, Yasir Bin Nisar, Jonathon Simon, Wilson Were

**Affiliations:** 1 Department of Paediatrics, University of Melbourne, Melbourne, Australia.; 2 Infection and Immunity Theme, Murdoch Children’s Research Institute, Royal Children’s Hospital, Parkville, Victoria, Australia.; 3 Royal Children’s Hospital Melbourne, Flemington Road, Parkville, Victoria, Australia; 4 Department of Infectious Disease Epidemiology, London School of Hygiene and Tropical Medicine, London, United Kingdom

## Abstract

**Background:**

Pneumonia is a major cause of death in children aged under five years. As children with severe pneumonia have the highest risk of morbidity and mortality, previous studies have evaluated the additional benefit of adjunctive treatments such as oseltamivir, oral steroids, macrolides, and vitamin supplementation that can be added to standard antibiotic management to improve clinical outcomes. The study reviewed the evidence for the role of these additional treatments for children with severe pneumonia in low- and middle-income countries (LMICs).

**Methods:**

Four electronic databases were searched for English-language articles between 2000 to 2020. Systematic reviews (SRs) with meta-analyses, comparative cohort studies, and randomised controlled trials (RCTs) from LMICs that reported clinical outcomes for children with severe pneumonia aged between one month to 9 years who received adjunct treatment in addition to standard care were included. Risk of bias of included SRs was assessed using AMSTAR 2, and of individual studies using the Effective Public Health Practice Project (EPHPP) quality assessment tool for quantitative studies.

**Results:**

Overall, the search identified 2147 articles, 32 of which were eligible, including 7 SRs and 25 RCTs. These studies evaluated zinc (4 SRs, 17 RCTs), Vitamin D (1 SR, 4 RCTs), Vitamin A (3 SRs, 1 RCT), Vitamin C (1 SR, 2 RCTs) and micronutrients (1 RCT). Most studies reported clinical outcomes of time to improvement, length of stay, and treatment failure (including mortality). No studies of oseltamivir, steroids, or macrolides fulfilling the inclusion criteria were identified. For zinc, pooled analyses from SRs showed no evidence of benefit. Similarly, a Cochrane review and one RCT found that Vitamin A did not improve clinical outcomes. For Vitamin D, an RCT evaluating a single high dose of 100 000 international units (IU) of vitamin D found a reduction in time to improvement, with 38%-40% documented vitamin D deficiency at baseline. However, two other studies of 1000 IU daily did not show any effect, but vitamin D status was not measured. For vitamin C, two studies found a reduction in time to symptom resolution in those with severe disease, with one reporting a shorter length of hospital stay. However, both studies were of weak quality. Most studies excluded malnourished children, and studies which included these children did not report specifically on the effect of micronutrients.

**Conclusions:**

This review found that adjunctive zinc and vitamin A, in addition to standard care, does not improve clinical outcomes in children with severe pneumonia in LMICs (strong evidence). However, a reduction in time to symptom resolution was reported with high dose vitamin D supplementation in children with documented vitamin D deficiency (strong evidence from one study) and vitamin C (weak evidence), although further research is needed, especially in underweight children.

Pneumonia is a major cause of death in children under five years of age, causing approximately one million deaths worldwide annually [1]. In 2015, 22 million (16%) of 138 million pneumonia cases internationally were classified as severe by WHO criteria [2]. The WHO Integrated Management of Childhood Illness (IMCI) criteria defines children with severe pneumonia as those with signs of pneumonia and at least one of the IMCI danger signs; not being fed well, convulsions, reduced consciousness level, reduced movement, fever (>38°C), or hypothermia (<35.5°C), and central cyanosis [3,4]. The case fatality rate for severe pneumonia is up to six times higher than for non-severe pneumonia (4.2% compared to 0.65%) [2]. Therefore, low cost, accessible adjunctive therapies to improve clinical outcomes are needed, particularly in younger children.

The current WHO IMCI recommendation for the first-line treatment of severe pneumonia for children aged 2 to 59 months is parenteral ampicillin (50mg/kg every six hours for at least five days) or penicillin, and gentamicin (7.5mg/kg once a day for at least five days) [5]. Some proposed additional therapies include vitamin supplementation such as zinc [6-8], vitamin A [9], vitamin D [10,11], and other micronutrients such as folic acid [12] due to the high rate of malnutrition in low- and middle-income countries (LMICs). Also, macrolide antibiotics are recommended for severe pneumonia in high-income countries to cover atypical bacteria – a recommendation that is not currently included in WHO guidelines [13]. Although diagnostic tests for influenza are not widely available in LMICs, influenza has been shown to frequently cause viral pneumonia in children. The role of neuraminidase inhibitors such as oseltamivir (Tamiflu) and zanamivir in addition to standard care for children with severe pneumonia in this setting has not been established. Finally, oral corticosteroids, along with azithromycin, have been associated with a reduction in the duration of hypoxemia and dyspnoea in children with pneumonia due to *Mycoplasma pneumoniae* in China [14].

Therefore, this review aims to explore the role of additional treatments with vitamins, neuraminidase inhibitors, macrolides, and oral steroids on clinical outcomes, specifically for children aged one month to nine years with severe pneumonia in LMICs.

## METHODS

### Data sources

MEDLINE, Embase, PubMed, and the Cochrane library were searched in September 2020. Authors of identified studies that were still recruiting or had an unclear recruitment status were contacted for information on study status and availability of results. References of relevant reviews and included studies were searched for additional studies.

### Search strategy

The search strategy included terms related to pneumonia (the condition of interest) and treatment outcomes. It also included terms related to specific additional therapies, including vitamin supplementation, oseltamivir, oral steroids, and macrolides. This included synonyms for these treatments, such as other medications within the same class and relevant specific names of vitamins. The search strategy also included an extensive list of terms related to the setting of interest (LMICs). See Appendix S1 in the **Online Supplementary Document** for the full MEDLINE search strategy.

### Eligibility criteria

Included studies involved children aged one month to nine years with severe pneumonia (by any definition) in LMICs according to World Bank income level. Studies that looked at children with severe pneumonia caused only by one specific pathogen such as measles or *M. pneumoniae* were excluded. Those studies reporting additional treatment with either vitamins, neuraminidase inhibitors (oseltamivir or zanamivir), macrolides or oral steroids, and that compared clinical outcomes to standard care (as per the study setting) were included. Systematic reviews (SRs), cohort studies, and clinical trials published in English between 2000 to the search date were included. Original studies were included and presented separately even when the results had been reported in the identified SRs. Literature reviews, case series, case reports, and conference, meeting, and poster abstracts were excluded.

### Study selection

Titles and/or abstracts of retrieved studies were screened by a single investigator (MHN) to identify studies meeting the inclusion criteria. Any queries about the studies’ inclusion eligibility were discussed with a second reviewer (AG). Separate standardised tables were used for data extraction. Extracted data included: study year, WHO region, study aim, population data (including age and standard treatment), definition of severe pneumonia, details of the additional treatment, baseline deficiency (for studies of nutritional supplements only), pathogens identified, and key clinical outcomes. For SRs, the extracted data included: Author, year, whether it was a Cochrane review, inclusion and exclusion criteria, outcomes, search results and analysis, study interventions, key clinical outcomes, and adverse events.

### Risk of bias in individual studies

One investigator (MHN) assessed the risk of bias of included SRs using AMSTAR 2 [15], while individual studies were assessed using the Effective Public Health Practice Project (EPHPP) quality assessment tool for quantitative studies [16].

A narrative synthesis of findings based on the results of included studies was planned.

## RESULTS

**Figure 1:**
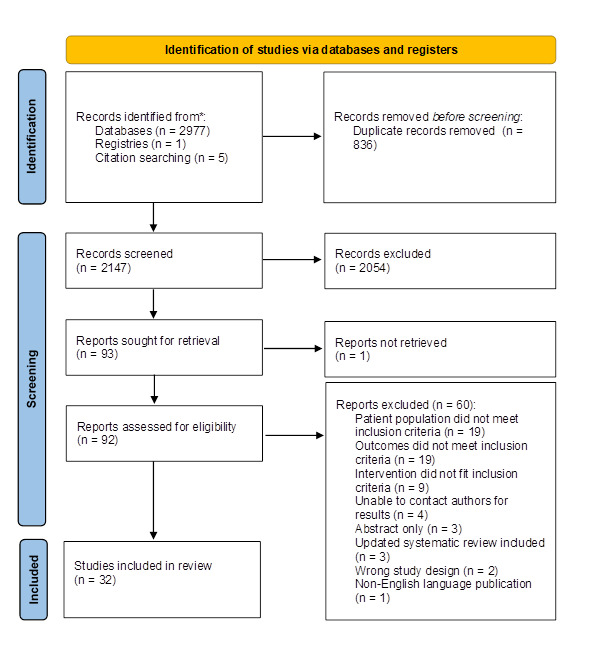
PRISMA Flow diagram for review.

2054 of 2147 identified studies were excluded after titles/abstracts were reviewed, leaving 93 full-text articles to be assessed for eligibility. We were unable to locate one of these full-text articles, and a further 60 were excluded based on eligibility criteria, leaving 32 studies for final inclusion. The most common exclusion reasons were the population (19 studies), outcomes (19 studies), and intervention (9 studies). Additionally, one study was identified after contacting the lead investigator for a trial identified from clinicaltrials.gov, and five were identified after a manual searching of all references (**Figure 1**).

### Study characteristics

**Table 1** summarises the included SRs for effectiveness of adjunctive therapies in children with severe pneumonia in LMICs. A total of 32 studies were identified, including seven SRs and 25 RCTs. All SRs studied nutritional supplements as an adjunct treatment of pneumonia, with two evaluating zinc [17,18] and one each evaluating vitamin A [20], vitamin D [23], and vitamin C [22]. Two of these SRs examined the effectiveness of multiple adjunct therapies (zinc and vitamin A) [19,20].

**Table 1:** Included systematic reviews for effectiveness of adjunctive therapies in children with severe pneumonia in LMICs

Review details	Review search parameters	Outcomes	Search results/ analysis	Intervention	Key clinical outcomes*			AEs	Quality
					**Time to improvement**	**Length of stay**	**Treatment failure**		
Brown 2020 [17]	**Aim:** To investigate the efficacy of adjunctive Zn supplementation in children aged 2-60m with pneumonia in LMICs. **Databases/websites searched:** MEDLINE, the Cochrane Library, EMBASE, LILACS, SciELO, the WHO portal, Scopus, Google Scholar and ClinicalTrials. gov. **Inclusion criteria:** RCTs where Zn given as an adjunctive Rx to std pneumonia therapy including AB **Exclusion criteria:** Studies with factorial designs or comparing Zn with another potentially active adjunctive Rx	**Primary:** 1) Treatment failure **Secondary:** 1) Time to recovery from pneumonia; 2) Mortality	11 RCTs: India (3); Bangladesh (1); Nepal (3); Tanzania (1); The Gambia (1); Ecuador (1); Uganda (1), n = 6497 children aged 2-60m. All studies included children with severe pneumonia, with 10 including only children with severe pneumonia. Meta-analysis including sensitivity analysis for bias performed.	>12m: 10 studies, Zn = 20mg OD; 1 study, Zn = 25mg OD <12m: 4 studies, Zn = 10mg OD; 5 studies, Zn = 20mg OD	7 studies included in pooled HR = 1.01, 95% CI = 0.89, 1.14	Treatment failure (primary): 10 studies included in pooled OR = 0.93, 95% CI = 0.75, 1.14	Mortality: 8 studies included in pooled OR = 0.64, 95% CI = 0.31, 1.31	-	Mod/High
Haider 2011 [18] Cochrane review	**Aim:** To evaluate Zn as an adjunct to AB in the treatment of pneumonia in children aged 2-59m **Databases/websites searched:** CENTRAL, MEDLINE, EMBASE, CINAHL, LILACS, AMED, CAB, Web of Science **Inclusion criteria:** RCTs evaluating adjunctive Zn with AB for pneumonia in children aged 2-59m **Exclusion criteria:** Non-RCTs; quasi-RCTs; studies of children with other debilitating diseases	**Primary outcomes:** 1) Time-to-clinical recovery **Secondary:** 1) Time-to-hospital discharge 2) Readmission/re-diagnosis with pneumonia 3) Mortality within 10d & 1m of randomization	4 RCTs: India (2); Bangladesh (1); Nepal (1), n = 3267 children All studies included children with severe pneumonia, with 3 including only children with severe pneumonia. Meta-analysis performed.	3 studies: Zn 20mg = OD for 5-15d1 study: Zn = 10mg for children aged 2-11m	2 studies included in pooled HR = 1.12, 95% CI = 0.89, 1.41	Time-to-hospital discharge: 3 studies included in Pooled HR = 1.04, 95% CI = 0.89, 1.22	-	No SAEs reported	Mod/High
Mathew 2011 [19]	**Aim:** To determine the therapeutic role of Zn and Vit A when given with AB in children with pneumonia **Databases searched:** Cochrane Library, MEDLINE through PubMed, IndMed, Popline.org, WHO & UNICEF, Government of India, Conference proceedings, abstract books **Inclusion criteria:** Not stated **Exclusion criteria:** Not stated	**Zinc** 1) Mortality 2) Length of hospitalization 3) Duration of illness 4) Complications 5) Side Effects	1 SR including 4 trials & 2 additional RCTs; 2 studies from RCT, and 1 additional RCT looked at children with severe pneumonia only, and the other studies included both non-severe and severe cases. Number of children not reported.	Dose not reported	‘A SR with 4 included trials suggested ‘no therapeutic benefit of adding zinc to AB therapy. Since then, two more trials have confirmed the absence of benefit in pneumonia as well as severe pneumonia’				Critically low
		**Vitamin A** As above	1 SR including 9 trials; 1 Cochrane review. Number of children not reported. Severity of pneumonia in studies not stated.	Dose not reported	“In the therapy trials, five outcomes of mortality, duration of hospitalization, duration of illness, complications, and side effects were not significantly different with Vit A or placebo.”				
Theodoratou 2010 [20]	**Aim:** Assess the effect of pneumonia case management (including Zn, Vit A supplementation) on mortality from childhood pneumonia and other secondary outcomes **Databases/websites searched:** MEDLINE, EMBASE, Web of Knowledge **Inclusion criteria:** RCTs, cRCTs, quasi-RCTs or observation studies Control arm of placebo or no Rx Children aged <5y old with clear case definition consistent with pneumonia No language or publication restrictions	**Zinc** 1) Length of hospitalization 2) Time to resolution	5 RCTs from Asia; 4 studies included children aged 2-24m, 1 included children 9m-15y with measles pneumonia (not included in pooled RR). Meta-analysis performed for any outcome with >1 study	Zinc sulfate and acetate	Severe illness: 2 RCTs included in pooled RR = 0.83, 95% CI = 0.48, 1.44. Low outcome specific quality	2 RCTs included in pooled RR = 0.87, 95% CI = 0.55, 1.37. Low outcome specific quality.		-	Critically low
		**Vitamin A** 1) All-cause mortality of children with pneumonia 2) Length of hospitalization	**Vit A:** 9 RCTs from Asia, Africa, S. America; 1 study included children aged 3-11m. Meta-analysis performed for any outcome with >1 study	In 2 RCTs Vit E included in Vit A supplement	Hypoxia: 4 studies included in WMD -0.02 (95% CI -0.16-0.12) Low outcome specific quality Tachypnoea: 5 studies included in WMD = 0.05, 95% CI = -0.21, 0.31 Low outcome specific quality	3 studies included in WMD = 0.04 (95% = -0.40, 0.48) Low outcome specific quality	Mortality 6 studies included in RR = 1.09,95% CI = 0.59, 2.04 Very low outcome specific quality	-	
Wu 2005 [21] Cochrane review	**Aim:** To determine whether adjunctive vit A is effective in children diagnosed with non-measles pneumonia **Databases/websites searched:** CENTRAL, MEDLINE, EMBASE, PubMed, LILACS, CINAHL, Biological Abstracts, Current Contents, and Chinese Biomedicine Database, WHO ICTRP **Inclusion criteria:** Parallel-arm RCTs, quasi-RCTs, in which children diagnosed with non-measles pneumonia were treated with Vit A Non-specific pneumonia that was uncomplicated by measles Pneumonia defined using clinical case definition, radiology, or both Vit A plus standard treatment vs standard treatment ± placebo **Exclusion criteria:** Studies of Vit A preventing non-measles pneumonia; studies including patients with measles	**Primary:** 1) Mortality **Secondary:** 1) Signs of pneumonia (eg, fever, tachypnoea) 2) Clinical severity 3) Adverse events	6 RCTs from LMICs: China (2); Tanzania (1); Brazil (1); Ecuador (1); Peru (1), n = 1740 children aged from 1m-14y. None specifically included children with only severe pneumonia. Method of analysis: Statistically combined results when appropriate	5 studies gave ≥100 000 IU of vitamin A to children ≥1y, and between 50 000 IU and 100 000 IU to ≤1y OD either as a once off or for 2d. 1 study: Vit A 1000 IU BD for 6d followed by 1500 IU/d for 20d	Time for remission of signs† with basal serum retinol concentration >200 μg/L, 1 study with outcomes for severe pneumonia: MD = -61.40, 95% CI = -119.10, -3.7. Quality not assessed for subgroup analysis.		Mortality during hospitalization, 1 study with severe pneumonia; OR = 1.61, 95% = CI 0.68, 3.83 Quality not assessed for subgroup analysis	3 studies: Vomiting (2 studies, OR = 0, 95% CI = 0, 1.33; Bulging fontanelle (1 study, OR = 0, 95% CI = 0, 155.37 Diarrhoea (1 study, OR = 0.57, 95% CI = 0.31, 1.05; irritability (1 study, OR = 0.93, 95% CI = 0.56, 1.57.	Mod/High
**Vitamin C**									
Padhani 2020 [22] Cochrane review	**Aim of review:** To assess the impact of vit C supplementation to prevent and treat pneumonia in children and adults **Databases and websites searched:** CENTRAL, MEDLINE, EMBASE, PubMED, LILACS, CINAHL, Web of Science, ClinicalTrials.gov, ICTRP **Inclusion criteria:** RCTs evaluating the role of vit C as an adjunct to pneumonia treatment compared to placebo No restrictions on language or publication status **Exclusion criteria:** Studies with participants with immune suppression, or with a primary diagnosis of meningitis, asthma, sickle cell anemia, HIV/AIDS, and severe malnutrition. Excluded those with ventilator-associated pneumonia or HAP	**Primary:** 1) Duration of illness 2) Clinical cure rate (defined as clinical recovery by the end of treatment) 3) Mortality due to pneumonia 4) Adverse effects	**Number of studies included:** 5 RCTs: 4 RCTs included children aged <5 y: Pakistan (2); Bangladesh (1); Chile (1), n = 1214 children <5y. 2 of these studies included children with severe pneumonia, and 2 did not specify the severity of pneumonia	Doses of Vit C were 125 mg, 200 mg (either BD for 4w, OD until symptoms improved, or every 8 h until discharge) and to 2 g (twice weekly for 12w).	Two studies reported on duration of illness but could not pool as used different measures. One study reported decrease in number of days for improvement in Sa02 (1.03d ±0.16 vs 1.14d ±1.0, *P* = 0.003) and Resp Rate (3.61d ±1.50 vs 4.04d ±1.62, *P* = 0.44) in the vit C compared to control group. One study reported a decrease in the duration of illness in the vit C group (3.4d ±2.54) vs control (4.5d ±2.35) (*P* = 0.038). *Severity of condition not specified.* Quality of evidence very low.	Two studies reported duration of hospitalization, but could not pool as one study did not report SD Both reported a lower mean hospital stay in the Vit C group vs control: 1 study, mean duration of hospital stay in Vit C group 6.75d vs 7.75d control group; 1 study, lower mean duration of stay of 109.55 h ±27.89 vs 130.64 h ±41.76 in the control group (*P* = 0.001). *Severity of condition not specified.* Quality of evidence very low	-	-	Mod/High
**Vit D**									
Das 2018 [23] Cochrane review	**Aim**: To evaluate the efficacy and safety of vitamin D supplementation as an adjunct to antibiotics for the treatment of acute childhood pneumonia. **Databases and websites searched:** CENTRAL, MEDLINE, EMBASE, ClinicalTrials.gov, ICTRP **Inclusion criteria:** RCTs including children (aged <1m & up to 5y) hospitalised with acute CAP, as defined by the WHO acute respiratory infection guidelines that compared vitamin D supplementation with control.	**Primary outcomes:** 1) Time to resolution of acute illness 2) Duration of hospitalization **Secondary** 1) Time to resolution of sx (tachypnea, indrawing, hypoxia, fever, inability to feed) 2) Treatment failure rate 3) Mortality rate 4) Adverse events	**Number of studies included:** 7 RCTs in LMICs: India (4); Afghanistan (1); Iran (1); Pakistan (1), n = 1529 children, 780 with pneumonia, 749 with severe or very severe pneumonia.	4 studies: single dose Vit D 100 000 IU; 2 studies: Vit D OD for 5 d (1000 IU if <1y; 2000 IU if ≥1y); 1 study: Vit D 50 000 IU OD for 2 d	3 studies included in MD = 0.95, 95% CI = -6.14, 4.24 Quality of evidence low.	4 studies included in MD = 0.49, 95% CI = -8.41, 9.4; 4 (2 of severe pneumonia). Quality of evidence very low	Mortality rate: 1 study included in RR = 0.97, 95% CI = 0.06, 15.28. Quality of evidence very low.	No major adverse events were reported	Mod/High

AE – adverse events, Zn - zinc, M – month, HR – hazard ratio, OR – odds ratio, CI - confidence interval, Mod – moderate, d – day, SAE – serious adverse events, Vit – vitamin, y – year, SR – systematic review, cRCT – cluster randomized controlled trial, WMD – weighted mean difference, RR – risk ratio, WHO – World Health Organization, ICTRP – International Clinical Trials Registry Platform, LMIC – low and middle-income country, IU – international units, OD – once daily, MD – mean difference, BD – twice daily, hr – hour, Sa02 – oxygen saturation, Resp Rate – respiratory rate, CAP – community acquired pneumonia, sx – symptoms

*Outcomes for children with severe pneumonia given unless otherwise stated.

†Tachypnoea, fever & hypoxemia.

**Table 2** shows the included individual studies for effectiveness of adjunctive therapies in children with severe pneumonia in LMICs. 25 studies identified were all RCTs; 17 examined the effect of zinc as an additional therapy, four evaluated vitamin D, and one studied vitamin A and vitamin C. Two studies evaluated combinations of different micronutrients, including a multi-nutrient containing vitamin A, C, E, folic acid, and zinc (one study) as well as a combination of vitamin C and E (one study). Most studies were from the Southeast Asia region (14 studies), with the remaining studies being from the Eastern Mediterranean (five studies), Americas (three studies), African (two studies), and Western Pacific region (one study). In 12 studies, the WHO definition of severe pneumonia was specifically used, in four studies the definition was not stated, and the remaining studies used alternative definitions. Most studies included young children aged 2-59 months, and in 18 of the 25 included studies, children with malnutrition were excluded.

**Table 2:** Included individual studies for effectiveness of adjunctive therapies in children with severe pneumonia in LMICs

Study, year, WHO region, Country	Study design	Study aim	Participants	Definition of severe pneumonia	Adjunct treatment and dose	Baseline deficiency		Pathogen	Key clinical outcomes			EPHPP
**Time to improvement**	**Length of stay**	**Treatment failure**	
**Zinc**												
Acevedo-Murillo 2019 [24] Region of the Americas, Mexico	Triple-blinded RCT	**Primary:** Effect of Zn on time to clinical improvement **Secondary:** Evaluate the immune-modulatory effect of zinc supplementation	n = 103 CAP • 53 AB only mean age = 23 ± 2.2m 53 AB only • 51 Zn + AB mean age = 18 ± 2.2m		Zinc sulfate <1y = 10mg, >1y = 20mg	Mean zinc level±SE (μmol/L); Placebo = 210 ± 19 Zinc = 230 ± 18		Viruses: RSV A & B, rhinovirus, HMPV Bacteria: *H. influenzae* & *S. pneumoniae*	Mean±SE (h): Placebo = 105 ± 8; Zinc = 76 ± 7; *P* = 0.01*	Mean±SE (d): Placebo = 4.8 ± 0.3; Zinc = 4 ± 0.2; *P* = 0.91	No deaths in either group	Strong
Bansal, 2011 [8] SE Asia, India	Triple-blind placebo-controlled RCT	**Primary:** Time to be asymptomatic **Secondary:** • Time to achieve Sao_2_>95% in RA • Time to disappearance of danger signs • Time to resolution of respiratory distress • Time to resolution of tachypnea • Length of stay	n = 106 CAP • 53 AB only median age = 4m (IQR = 3-12) • 52 Zn + AB median = 6m (IQR = 2.7-9)	Tachypnea and chest indrawing or one of the danger signs†	Zinc gluconate 20mg OD for 5d	Similar baseline level in both groups		-	Median, IQR (h): Placebo = 53, 30-72 Zinc = 60, 24-78 *P* = 0.98	Median, IQR (d): Placebo = 5, 3-6.5 Zinc = 5, 4-5.5 *P* = 0.63	**-**	Strong
Baruah 2018 [25] SE Asia, India	Randomized, double-blind, placebo-controlled trial	**Primary:** 1) Time for clinical resolution of pneumonia **Secondary:** 1) Hospital stay 2) Recurrence of pneumonia in next 3m	n = 560 CAP • 280 AB only mean age ±SD = 9.8 ± 9.5m • 51 Zn + AB Mean age ±SD = 9.3 ± 9.9m	WHO	Zinc gluconate for 2w: 2-6m = 10mg OD 7-60m = 20 mg OD	Mean zinc level±SD (μmol/L): Placebo = 21.3 ± 10.1 Zinc = 21.3 ± 7.3		-	<3d for clinical resolution of pneumonia (primary) Placebo = 69/280 (25%) Zinc = 91/280 (33%) OR = 0.68 (95% CI = 0.47, 0.98) *P* = 0.040*	<3d hospital stay: Placebo = 127/280 (45%) Zinc = 152/280 (54%) OR = 0.70 (95% CI = 0.50, 0.97) *P* = 0.035*	**-**	Mod
Basnet 2012 [26] SE Asia, Nepal	Double-blind placebo-controlled RCT	**Primary**: Median time to cessation of severe pneumonia in hours **Secondary outcomes** • Proportion with duration of severe pneumonia in hours • Proportion with treatment failure	n = 598 CAP • 299 AB only mean age = 7.1 ± 5.6m • 299 Zn + AB (intervention) mean age = 7.8 ± 6.0m	WHO	Zinc sulfate OD until discharge or max of 14d <12m = 10mg ≥12m = 20mg	-		Viruses in 29%	Median, IQR (h) Placebo = 49, 29-91 Zinc = 49, 33-77 HR = 1.10 (95% CI = 0.94, 1.30)		Placebo = 298/299 (99.7%) Zinc = 296/299 (99.0%) HR = 0.88 (95% CI = 0.71, 1.10)	Strong
Bose, 2006 [6] SE Asia, India	Double-blind Placebo RCT	**Primary outcomes:** 1) Time to resolution of severe pneumonia 2) Duration of hospitalization	n = 300 CAP • 150 AB only, mean age 9.1 ± 5.7m • 150 Zn + AB, mean age = 9.9 ± 6.1m	RR>50/min *and* crepitations on auscultation *and* presence of ≥1 danger signs	Zinc sulfate d0 = 20mg STAT d2-14 = 10mg BD	Mean zinc level±SE (μmol/L): Placebo = 10.9 ± 2.4 Zinc = 11.0 ± 2.2 μmol/L Difference between groups were not significant by Student’s *t* test		-	Median‡, 95% CI (h): Placebo = 96.7; 78.2-112.9 Zinc = 111.3; 88.5-138.0 RR = 0.86; 95% CI = 0.62, 1.18) *P* = 0.35	Median, 95% CI (h): Placebo: 72.3; 67.7-79.6 Zinc = 71.1; 68.1- 87.3 RR = 0.93; 95% CI = (0.74, 1.17) *P* = 0.55		Strong
Brooks 2004 [7] SE Asia, Bangladesh	Double-blind placebo-controlled RCT	**Primary outcome:** 1) Time to cessation of severe pneumonia§ 2) Discharge from hospital	n = 270 CAP • 135 AB only, mean age = 9.6 ± 6.0m • 135 Zinc AB (intervention), mean age = 9.5 ± 6.2m	Pneumonia and either chest indrawing or ≥1 danger signs	20mg PO OD until discharged from hospital	Mean zinc level±SE (μmol/L): Placebo = 10.1 ± 1.0 Zinc = 10.1 ± 1.1μmol/		NS	Severe pneumonia resolution Median, 95% CI (h): Placebo = 96; 72-96 Zinc = 72; 72-96 RR = 0.70, 95% CI = 0.51, 0.98*	Median, 95% CI (h): Placebo = 112; 111-129 Zinc = 112; 104-112 RR = 0.75, 95% CI = 0.57, 0.99*		Mod
Sempertegui 2014 [27] Region of the Americas, Ecuador	Double-blind, placebo-RCT	**Primary:** Resolution of respiratory signs **Secondary:** Treatment failure	n = 550 CAP • 255 AB only, mean age 13.0 ± 11.2m • 225 Zn + AB, mean age 13.1 ± 10.3m	Modified WHO criteria^‖^	Zinc sulfate 20mg BD while in hospital	Mean zinc level±SE (μmol/L): Placebo = 7.4 ± 2.5 **L** Zinc = 7.6 ± 2.7 **L**		Viruses: RSV = 39.2%; hMPV = 17.5%; adenovirus = 15.3% Bacteria: *S. pneumoniae* (9.2%) Pathogens isolated from plasma and NP samples	Mean±SE (h): Placebo (n = 178) = 93.9 ± 9.8 Zinc (n = 191): 102.6 ± 76.1 *P*-values nonsignificant (Student’s *t* test)	-	Placebo = 76/221 (34%) Zinc: 76/220 (34.5%) OR = 1.00, 95% CI = 0.68, 1.50	Strong
Wadhwa 2013 [28] SE Asia, India	Double-blind, placebo-RCT	**Primary:** Time from random assignment until recovery **Secondary:** Treatment failure	n = 550 CAP • 276 AB only, median age 5m (IQR 3-10) • 274 Zn + AB, median age = 5.5m (IQR = 3-10)	WHO criteria	Zinc sulfate 10mg BD until recovery or 14d course.	Mean zinc level±SD (μmol/L): Placebo = 9.2 ± 3.6 **L** Zinc group: 9.3 ± 3.9 **L**		-	Median, IQR (h): Placebo = 77.0, 58-117 Zinc = 78.5, 59-122 HR = 0.98, 95% CI = 0.82, 1.17	-	Placebo = 28/263 (10.6%) Zinc grp = 37/262 (14.1%) RR = 1.3, 95% CI = 0.8, 2.1 Death Placebo = 4/276 (1.5%) Zinc grp = 4/274 (1.5%) RR = 1.0, 95% CI = 0.3, 4.0	Strong
Hashemian 2020 [29] Eastern Mediterranean, Iran	Double-blind placebo-controlled clinical trial	**Primary:** Recovery time for fever and tachypnea **Secondary:** Duration of hospitalization and mortality rate	n = 120 CAP • 60 AB only, mean age = 12.7 ± 10.4m • 60 Zn + AB, mean age 16.7 ± 15.1	Pneumonia and sx of respiratory distress eg, Tachypnea or chest retraction.	Zinc sulfate OD for 7d <1y = 10mg ≥1y = 20mg	-		-	Mean±SD time for Resp Rate to normalize (d) (primary): Placebo = 2.1 ± 0.8 Zinc = 1.8 ± 0.8 *P* = 0.011*	Mean±SD (d): Placebo = 7.2 ± 1.2 Zinc = 7.1 ± 1.2 *P* = 0.728		Mod
Howie 2018 [30] Africa, Gambia	Double-blind, placebo-RCT	**Primary:** Prevention of ‘treatment failure’ (presence of any sign of severe pneumonia on d5 and d10) **Secondary:** Time to resolution of signs of severe pneumonia (secondary)	n = 604 CAP • 301 IV AB only, median age = 13m (IQR = 7-24) • 303 Zinc + AB, median age = 13 (IQR = 6-23)	Modified WHO criteria	Zinc sulfate OD for 7d <1y = 10mg ≥1y = 20mg	Median (IQR) Zinc level (μmol/L): Placebo = 14.0 (7.5-23.7) Zinc = 11.3, (7.6-19.4)		-	Median (h): Placebo (n = 36) = 42.3 Zinc (n = 31) = 30.9 OR = 0.81, 95% CI = 0.58, 1.15 *P* = 0.242	Median (h): Placebo (n = 292) = 95.9 Zinc (n = 296) = 94.7 (296) OR = 1.04, 95% CI = 0.94, 1.15 *P* = 0.468	Day-5 Placebo = 41/293 (14.9%) Zinc = 42/298 (14.1%) OR (adjusted) = 1.08, 95% CI = 0.65, 1.80) *P* = 0.773	Strong
Laghari 2019 [31] Eastern Mediterranean, Pakistan	RCT	**Primary:** Alleviating symptoms and shortening of hospital stay	n = 100 CAP • 50 AB only, mean age 30 ± 4m • 50 Zinc + AB mean age 27 ± 6m	Pneumonia *and*≥1 danger sign	Zinc 20mg OD	-		-	-	Mean±SD (h): Placebo (n = 178) = 3.57 ± 0.81 Zinc (n = 191) = 3.12 ± 0.99 *P* = 0.01*	-	Weak
Manohar 2015 [32] SE Asia, India	Double-blind placebo-controlled RCT	• Time to reach Sa02 > 90% in RA • Length of hospital stay • Nil per oral duration • Treatment failure requiring 2nd and 3rd line AB Primary outcome NS	n = 110 CAP • 54 Zinc + AB, mean age 26.7 ± 16.5m 53 AB only, mean age = 28.3 ± 14.3m	WHO criteria	Elemental zinc 20mg OD for 14d	-		-	Time to reach Sa02 > 90% in RA (h) Mean±SD: Placebo = 49.4 ± 24.7 Zinc = 34.1 ± 19.7 *P* = 0.009*	Mean±SD (d): Placebo = 8.9 ± 3.12 Zinc = 7.2 ± 1.95 *P* = 0.001*	Fewer in zinc grp requiring 2nd or 3rd line AB (numbers not stated) *P* = 0.016*	Weak
Shah 2012 [33] SE Asia, Nepal	Double-blind placebo- controlled RCT	**Primary:** Decrease in duration of severe pneumonia and pneumonia **Secondary:** 1) Decrease in duration of: • Nil per orally • IV fluids • Use of oxygen 2) Treatment failures requiring 2nd and 3rd line AB	n = 117 CAP • 53 AB only, median age = 10y, IQR = (6.0-18.5) • 64 Zinc + AB, median age = 9y (IQR = 5.0-14.7)	WHO criteria	Zinc sulfate d0-1 = 20mg STAT d2-7 = 10mg BD	-		-	Duration of severe pneumonia Median (IQR) (h): Placebo (n = 53) = 26 (16.0-46.0) Zinc (n = 64) = 34.2 (21.0-48.0) *P* = 0.22	Median (IQR) (h): Placebo (n = 53) = 72 (48.0-87.7) Zinc (n = 64) = 73.5 (49.5-107.5) *P* = 0.19	Requiring 2nd line AB: Placebo = 15/53 (28.3%) Zinc = 14/64 (21.9%) *P* = 0.42	Mod
Srinivasan 2012 [34] African, Uganda	Double-blind, placebo RCT	**Primary:** 1) Time taken for normalization of: Resp Rate; temperature 2) Time taken to reach Sao_2_≥92% **Secondary:** Proportion of children who died	n = 352 CAP • 176 AB only, median age = 10y (IQR = 6.0-18.5) • 176 Zinc + AB, median age = 9y (IQR = 5.0-14.7)	WHO criteria	Zinc OD for 7 d: <1y = 10mg ≥1y = 20mg	Median, IQR zinc level (μmol/L): Placebo = 4.8 (2.3-10.4) **L** Zinc = 4.4 (1.3-8.0) **L**		45/184 (24.5%) positive BC: *S. aureus* (16); *S. pneumoniae* (14*); L. monocytogenes* (3); *H. influenzae* (2)	Time to normalization of Resp Rate (primary) Median; 95% CI (h): Placebo = 86.0, 95% CI = 75.4, 96.6 Zinc = 96.0; 95% CI = 83.0-109.0 HR = 0.88, 95% CI = 0.69-1.13 *P* = 0.31		Case fatality rate: Placebo = 21/176 (11.9%) Zinc = 7/176 (4.0%) RR = 0.33, 95% CI = 0.15, 0.76*	Strong
Valavi 2011 [35] Eastern Mediterranean, Iran	Double-blind placebo controlled RCT	**Primary:** Time taken for clinical symptoms of severe pneumonia to resolve **Secondary:** Length of hospital stay	n = 128 CAP • 64 AB only age = 15.9m • 64 Zinc +AB age = 15.4m	Tachypnea and fever, crepitations during inspiration, findings of pneumonia on CXR, *and*≥1 danger sign	Zinc sulfate 1mg/kg/d (max 10mg) BD for 5d	-		-	Mean time for resolving all symptoms (h) (primary): Zinc = 42.3 Placebo = 47.5 *P* < 0.001*	Mean stay in hospital (h) (secondary): Placebo = 137.7 Zinc = 126.7 *P* < 0.001*		Strong
Valentiner-Branth 2010 [36] SE Asia, Nepal	Double-blind, placebo controlled RCT	**Primary:** 1) Treatment failure (defined as a need for change in antibiotics or hospitalization) 2) Time to recovery from pneumonia	n = 149 severe pneumonia CAP • 74 Zinc + AB 2-11m = 55/74 (74%) ≥12m = 19/74 (26%) 75 IV AB only (control) 2-11m = 56/75 (75%) ≥12m = 19/75 (25%)	WHO criteria	Zinc OD for 14d2: 11m = 10mg ≥12m: 20mg	Mean zinc level (μmol/L): Placebo = 2-11m - 8.9 ± 2.2 **L** ≥12m - 7.9 ± 1.5 **L** Zinc = 2-11m - 8.1 ± 2.9 **L** ≥12m - 13 ± 13		Viruses: Placebo: 2-11m = 51% ≥12m = 44% Zinc = 2-11m 39% ≥12m = 44%	Median time, IQR (d) to recovery (primary): HR (pooled for all age grps) = 1.1; 95% CI = 0.77, 1.5)	Median time to discharge from hospital (d): HR (pooled for all age grps) = 1.1; 95% CI 0.77, 1.5	Proportion treatment failure (primary) OR (pooled for all age grps) = 0.97, 95% CI = 0.42, 2.2	Strong
Yuan 2016 [37] Western Pacific, China	Non-randomized RCT	Determine serum Zn levels among children <1y of age with severe CAP Observe changes in serum Zn levels after Zn supplementation & whether these changes influence clinical outcomes of critically ill infants with CAP	n = 73 CAP • mean age = 2.0 ± 2.0m • 39 Zinc + AB • 34IV AB only (control)¶	NS, but in Pediatric Intensive Care Unit	Licorice zinc: <12m = 10mg OD >12m = 20mg OD	Mean zinc level (μmol/L): Placebo = 42.6 ± 9.3 **L** Zinc = 40.8 ± 8.5 **L**		**Overall** Bacteria = 31/73 (43%) Virus = 5/73 (7%) Others = 8/73 (11%) Unknown = 29/73 (40%)		Mean stay in hospital±SD (d): Placebo = 7.0 ± 4.0 Zinc = 9.0 ± 6.0		Mod
**Vitamins A, C, D**												
Rodriguez 2005 [9] Region of the Americas, Ecuador	Double-blind, placebo-controlled RCT	**Primary:** 1) Time to remission of all 3 signs of tachypnea, fever, and hypoxemia 2)Duration of hospitalization	n = 287 severe pneumonia CAP • 142 AB only, mean age 15.5 ± 13.2m • 145 **Vit A** + AB, mean age = 14.2 ± 10.4m	WHO criteria	2-12m Vit A: 50 000 IU >12-59m: 100 000 IU	Mean retinol (μg/L): Control = 162 ± 70.2 Vit A: 152 ± 64.5		-	Mean±SD time to remission of all 3 signs (h): Placebo = 114.7 ± 107.5 Vit A = 106.7 ± 79.0 Difference between group not significant, *P*-value: NS	Children with Sa02 < 80% in the placebo group spent 25h less time in hospital than those in vit A. *P* = 0.1	**-**	Strong
Choudhary 2012 [10] SE Asia, India	Double-blind placebo RCT	**Primary**: Time to resolution **Secondary:** 1) Length of stay and 2) time to resolution of tachypnea, chest retractions and inability to feed.	n = 200 CAP • 100 AB only Mean age = 13.8 ± 11.4m • 100 **Vit D** + AB Mean age = 14.1 ± 12.2m	Pneumonia *and* chest indrawing *or*≥1 danger sign	Vit D OD for 5 d║: <1y = 1000 IU 1-5y = 2000 IU	Evidence of rickets: Control = 3/100 (3%) Vit D = 2/100 (2%)		-	Median, IQR (h): Placebo = 64, 48-88 Vit D = 72, 48-96 *P* = 0.33	Median, IQR (h): Placebo = 104, 88-128 Vit D = 112, 96-136 *P* = 0.29	**-**	Mod
Gupta 2016 [38] SE Asia, India	Double-blind placebo-controlled RCT	**Primary:** 1) Time to resolution 2) Proportion of children having a recurrence of pneumonia in next 6 mo.	n = 324 CAP • 162 AB only Mean age = 16.9 ± 13.4m • 162 **Vit D** + AB Mean age = 16.4 ± 12.9m	Presence of lower chest indrawing in children presenting with cough or difficult breathing	Single dose Vit D = 100 000 IU PO	Serum 25(OH)D<12 ng/mL Control = 65/162 (40.1%) Vit D = 61/162 (37.6%)		-	Median; 95% CI (h): Placebo = 31; 95% CI = 29-33 Vit D = 30; 29-31 HR (unadjusted) = 1.31, 95% CI = 1.04, 1.64, *P* = 0.020 HR (adjusted) = 1.39, 95% CI = 1.11, 1.76; *P* = 0.005*			Strong
Manaseki-Holland 2010 [11] Eastern Mediterranean, Afghanistan	Double-blind placebo RCTs	**Primary:** 1) Duration of illness 2) Risk of repeat episodes of pneumonia over following 3m.	n = 453 CAP, 74 with severe pneumonia • 229 AB only, mean age = 13.19 ± 9.2m • 224 **Vit D** + AB mean age = 13.2 ± 9.1m. Of these 39 (17%) severe	WHO criteria (1995)	Vit D3 = 100 000 IU	In 2005 among 108 children aged 6-48m in Kabul = 73% significantly deficient		-	For all participants (pneumonia or severe pneumonia) Mean±SD (d): Placebo = 4.98 ± 2.89 Vit D = 4.74 ± 2.22 *P* = 0.2	Recovery within 24 h of admission** Vit D = 13/224 (6%) Placebo = 11/229 (5%) *P* = 0.68		Strong
Rajshekhar 2016 [39] SE Asia, India	Single-blinded RCT	**Primary:** Time to resolution of severe pneumonia **Secondary:** Duration of hospitalization	n = 96 CAP • 48 IV AB only, mean age = 2.08 ± 1.92y • 48 **Vit D** + AB, mean age = 1.94 ± 1.46y	WHO criteria	Vit D OD for 5d <1y = 1000 IU >1y = 2000 IU	NS serum levels.		-	Time taken for severe symptoms to subside 1)<24h Placebo = 30 (63%) Vit D = 10 (21%) 2) 24-48h Placebo = 28 (59%) Vit D = 30 (63%) 3)>48h Placebo = 15 (31%) Vit D = 30 (63%) *P* = 0.14			Weak
Mahalanabis 2006 [40] SE Asia, India	Double-blind placebo-controlled RCT	**Primary:** Evaluate the role of vit E and vit C as adjunct therapy of pneumonia in children	n = 174 CAP • 85 IV AB only, mean age = 10.0 ± 7.5m • 89 Vit E & C + AB, mean age = 8.8 ± 6.7m	ALRI or 1 severity indicator (not able to drink or feed, lethargy, irritability, nasal flare, drowsiness)	α-tocopherol (Vit E) 200mg & ascorbic acid (Vit C) 100mg PO BD for 5d	α-tocopherol (Vit E) Placebo = 10.25 ± 5.03 Vit E&C = 10.18 ± 4.60 *P* = 0.15		-	Effect on Illness indicators: 1) Very ill RR = 0.89, 95% CI = 0.64, 1.25, *P* = 0.5 2) Feeding difficulty RR = 1.01, 95% CI = 0.72, 1.41, *P* = 0.95 3) Tachypnoea RR = 1.12, 95% CI = 0.77, 1.64, *P* = 0.56	-	-	Strong
Khan 2014 [41] Eastern Mediterranean	Descriptive study	To determine the efficacy of vitamin C in reducing duration of severe pneumonia.	n = 222 CAP • 111 Vit C + AB aged: <1y = 53%; 1-3y = 34%; 4-5y = 13%. • 111 AB only (control) aged: <1y = 64%; 1-3y = 25%; 4-5y = 11%	NS	200mg of vitamin C OD	NS		-	<4d for Resp Rate to improve Placebo = 69/111 (62%) Vit C = 84/111 (76%) *P* = 0.03* <2d for chest indrawing to improve Placebo = 59/111 (53%) Vit C = 70/111 (63%) *P* = 0.14 <1d for hypoxia to improve Placebo = 96/111 (86%) Vit C = 108/111 (97%) *P* < 0.01*			Weak
**Micronutrient**												
Wahed 2008 [12] SE Asia, Bangladesh	RCT	**Primary:** 1) severity of pneumonia 2) duration of hospital stay	n = 800. Type of pneumonia NS, mean age = 6.5 ± 5.6m • 400 AB only • 400 micronutrient + AB††	Not stated	All or one of: Vit A, C, E, folic acid, zinc, dose NS Vit A = 0.60 ± 0.05 μmol/L Vit C = 32.50 ± 0.15 μmol/L Folic acid = 3.50 ± 0.04 nmol/L Vit C = 9.70 ± 0.74 μmol/L	Serum levels	-		Time (d) to resolution of‡‡: 1) Feeding difficulty Control = 2.0 Micronutrient = 2.0 2) Tachypnea: Control = 4.5 Micronutrient = 4.0 3) Chest indrawing: Control = 3.5 Micronutrient = 3.0 *P* < 0.01*	Mean (d) Control = 7.8 Micronutrient = 6.8 Average difference = 12.9% *P* < 0.01*		Weak

SE Asia – South-East Asia region, AB – antibiotics, BD – twice daily, IV – intravenous, OD – once daily, PO – *per os* (taken orally), STAT – immediately, Mod – moderate, BC – blood culture, CAP – community acquired pneumonia, d – day, grps – group, h – hour, m – months, NP – nasopharyngeal, NS – not stated, Pt – patient, RCT – randomised control trial, RA – room air, Resp Rate – respiratory rate, Vit – vitamin, w – week, WHO, World Health Organisation, y – year, CXR – chest x-ray, RA – room air, CI – confidence interval, Diff – difference, HR – hazard ratio, IQR – Interquartile range, RR – risk ratio, SD – standard deviation, SE – standard error, OD – odds ratio, L – low

When results were below lower limits of normal zinc reference range. Normal reference range based on using highest value for males aged 3-9 years, morning nonfasting (65, standard error 0.7 μg/dL; 9.94 ± 0.11 μmol/L) [42].

*Used to indicate strong evidence for outcome based on statistical testing where *P* value <0.05 or CI did not include the null value.

†Danger signs: Cyanosis, inability to feed/drink, lethargy and convulsions.

‡Using Definition 2 (Chest indrawing, O_2_ saturation <93%, RR>50) from article, as this definition closest to the WHO’s definition of severe pneumonia (tachypnoea +/− chest indrawing) + danger sign.

§No chest indrawing, respiratory rate ≤50 bpm, SaO_2_ ≥ 95% on RA.

¶Modified definition: Including the presence of cough and/or chest wall indrawing, tachypnoea (>50 bpm in 2-<12, >40 bpm in children 12-59 months), hypoxemia (SpO_2_ < 90%), and ≥ one of the following by auscultation: rales, diminished breath sounds, bronchial breath sounds, or pleural rub.

║Participants with clinical rickets were given a mega-dose of Vitamin D (6 000 000 IU).

**For all participants (pneumonia or severe pneumonia), and also recorded those for which date of recovery was not recorded.

††Micronutrient group: 200 children given all micronutrients (Vitamin A, C, E, folic acid, zinc), remaining 200 divided into 5 groups of 40 and given a specific treatment.

‡‡Summary average statistic used not stated.

### Zinc

The four SRs examining the effect of zinc supplementation as an adjunct to antibiotic treatment for severe pneumonia found no evidence of clinical benefit. Two of these studies were of moderate to high quality, and two were of critically low quality. The most recent SR published in 2020 included 11 RCTs involving 6497 children aged 2-60 months [17]. In the pooled analysis of 10 studies, the addition of zinc provided no protective effect against treatment failure (pooled OR = 0.93, 95% CI = 0.75, 1.14) nor a reduction in time to clinical improvement (7 RCTs, Pooled HR = 1.01, 95% CI = 0.89, 1.14). This SR [17] included all the original studies identified in the three previous SRs except for two studies [8,40]. One of these studies, an RCT including 106 children, showed no evidence for the benefit of zinc on clinical outcomes [8]. The data from this trial were included in the meta-analysis [18] that found zinc had no significant effect on time to clinical improvement (Pooled HR = 1.12; 95% CI = 0.89, 1.41) or length of stay (Pooled HR = 1.04; 95% CI = 0.89, 1.22). One study focused only on children with measles-related pneumonia, and therefore this study did not fulfil inclusion criteria for this review [18]. None of the included SRs did a sub-analysis for those children with baseline malnutrition. Notably, no SR reported serious adverse events associated with zinc treatment.

Of the 17 original studies, 6/15 RCTs showed a significant improvement in time to resolution of symptoms [7,24,25,29,32,35] and 5/13 RCTs showed a reduced length of hospital stay [7,25,31,32,35]. One of three RCTs reporting on death [34] showed that zinc reduced mortality by 67% (RR = 0.33; 95% CI = 0.15, 0.76) [34] while the other two found no difference (RR = 1.0; 95% CI = 0.3, 4.0) [28] and no deaths in either group [24]. Notably, this study [34] did not exclude those with malnutrition, and 26% of the zinc group and 25% of the placebo group were stunted at baseline (height-for-age z-score less than 2 standard deviations). This was also one of five studies where the enrolled children had baseline zinc deficiency [27,28,34,36,37] but was the only one of these studies that showed strong evidence for the clinical benefit of adjunctive zinc therapy [34]. Another study found no association between zinc supplementation and risk of death (RR = 1.0, 95% CI = 0.3, 4.0) despite severely underweight children comprising 21% and 25% of the zinc and placebo groups, respectively [28]. The remaining three studies [27,36,37] included children with baseline zinc deficiency and similarly found no evidence that zinc improved clinical outcomes, with one study showing an increased length of hospital stay in those treated with zinc compared to the placebo group (zinc = 9.0 ± 6.0 days vs placebo = 7.0 ± 4.0 days) [37]. These differing outcomes are not explained by variations in zinc dosing, given that 16 studies used doses of 10 to 20 mg per day.

## Vitamins

### Vitamin A

Three SRs in total studied the effect of vitamin A in children with pneumonia [19-21], with two looking at multiple outcomes of both vitamin A and zinc [19,20]. Both studies of vitamin A and zinc were of critically low quality [19,20], and the third SR was of moderate to high quality [21]. One SR identified 9 RCTs and performed a meta-analysis for the duration of hypoxia (4 RCTs), duration of tachypnoea (5 RCTs), length of stay (3 RCTs), and mortality (6 RCTs) [20]. These analyses found no difference between the vitamin A and placebo groups in any of these domains [20]. This SR primarily included children with non-measles pneumonia, as four of the six studies excluded measles pneumonia. Also, only one of the included studies [9] included outcomes specifically for children with severe pneumonia. Similarly, a Cochrane review of vitamin A on outcomes of non-measles pneumonia [21] included the same RCT reporting on children with severe pneumonia [9]. Another SR [19] reported no significant differences between Vitamin A groups and placebo for outcomes of mortality, duration of hospitalization, illness, and complications and side effects. There were no further original studies identified in this review since the publication of these systematic reviews.

Adverse effects were reported in three of the six included studies in the Cochrane review. A pooled analysis from two studies showed no association between vitamin A and vomiting (two studies, OR = 0.77; 95% CI = 0.45, 1.33), diarrhoea (one study, OR = 0.57; 95% CI = 0.31, 1.05), bulging fontanelles (one study, OR = 8.25; 95% CI = 0.44, 155.37), or irritability (one study, OR = 0.93; 95% CI = 0.56, 1.57) [21].

#### Vitamin D

A moderate to high quality Cochrane review of vitamin D in addition to antibiotic treatment for children with pneumonia identified 7 RCTs involving a total of 1529 children, with 749 (49%) having severe or very severe pneumonia [23]. A meta-analysis showed no clinical benefit of adjunct vitamin D on the duration of illness or hospitalization as well as mortality. Vitamin D doses varied between 1000 IU to 100 000 IU, and a subset analysis was not done based on dose. No serious adverse events were reported [23].

A double-blind placebo-controlled RCT provided strong evidence that a single dose of vitamin D 100 000 IU orally reduced the time to resolution of severe pneumonia (Adjusted HR = 1.39; 95% CI = 1.11, 1.76) [38]. However, three other RCTs showed no clinical benefit [10,11,39]. Among these, two studies [10,39] used low-dose vitamin D 1000 to 2000 IU/d for five days in children with severe pneumonia, and in the other study [11] that used a single dose of vitamin D3 100 000 IU, only 74 of 453 (16%) included children had severe pneumonia, which may explain the differing results. None of these studies reported any serious adverse events; however, one study reported one episode of vomiting and one of diarrhoea in the vitamin D group (1%) [10]. Only one study [38] reported baseline serum vitamin D deficiency, where deficiency (defined as serum 25(OH)D<12 ng/mL) was observed in 40% of the control group and 38% of the vitamin D group.

#### Vitamin C

A recent Cochrane review [22] included five RCTs of children under five years of age mostly from LMICs. However, the results of the studies could not be pooled due to different effect measures and data provided. This Cochrane review was assessed as being of moderate to high quality. Two of the studies included were published before the year 2000 [43,44], and one did not enrol children with severe pneumonia [45]. Both were excluded from this current review. One study from the Eastern Mediterranean region showed that a greater proportion of children treated with vitamin C had resolution of tachypnoea in less than four days and improvement in hypoxia in less than one day compared with placebo (tachypnoea = 76% vs 62%, *P* = 0.03; hypoxia = 97% vs 86%, *P* = <0.01) [41]. However, this study was assessed as being of poor quality due to a lack of description of randomization, and key differences between the groups at baseline not being adjusted for in the analysis. Similar results were found in an RCT of children in Southeast Asia that reported a significant reduction in the time to resolution of tachypnoea and chest indrawing by 0.5 days, and a 12.9% reduction in length of stay when children were treated with five micronutrients including vitamin A, C, and E, folic acid, and zinc [12]. This study was also of weak quality due to a lack of reporting on group baseline characteristics and giving a poor explanation for children not included in the analysis. There was only one study [12] that reported baseline serum vitamin C levels, with the average baseline serum concentration from all participants (n = 800) being just below the lower limit of normal (mean baseline serum vitamin C concentration = 32.50 ± 0.15 (standard deviation (SD)) μmol/L, lower limit of normal = 34.00 ± 113.00 μmol/L). Neither of these studies reported any adverse events [12,41].

### Combined nutritional supplements

There was no evidence from one high-quality RCT [40] for the addition of vitamin C and E to antibiotic treatment in symptom resolution (feeding difficulty: RR = 1.01, 95% CI = 0.72, 1.41; tachypnoea: RR = 1.12, 95% CI = 0.77, 1.64). This study recorded the mean weight for age z-score as being -1.78 (SD = ±1.18) in the control group and -1.92 (SD = ±1.18) in the intervention group, respectively, and excluded children with obvious marasmus or oedema [40].

## DISCUSSION

This review identified studies evaluating the clinical effectiveness of adding zinc, vitamins A, C, D, E, and micronutrients to antibiotic treatment in children with severe pneumonia in LMICs. We have found that adjunctive zinc and vitamin A do not improve clinical outcomes such as time to resolution of symptoms and length of hospital stay. For vitamin D, a meta-analysis showed no clinical benefit of adjunctive vitamin D. However, the results for low dose regimens (1000-2000 IU/d) and a single high dose (100 000 IU) were pooled. Of the two high-quality RCTs of high dose vitamin D identified in this review, the larger study [38] included 324 children with severe pneumonia and found an improvement in the time to resolution of symptoms in the context of a high proportion of deficiency at baseline, while the other study [11] which included only 74 children with severe pneumonia and unknown vitamin D status at baseline, found no benefit. It is possible that this beneficial effect for vitamin D on time to resolution of symptoms may be greater for those with vitamin D deficiency; however, it is difficult to assess this based on the limited evidence presented in this study [11], as it did not record vitamin D at baseline, but rather referenced previous surveys showing high proportions of vitamin D deficiency in Kabul, where the study was set [11]. Vitamin D has an important role in immune function through effects on the innate immune system such as engagement of toll-like receptors [46], and therefore high doses may be beneficial in boosting the immune response. A beneficial effect has been reported in a study of children with tuberculosis that showed faster symptom resolution in participants who received adjunctive vitamin D [47]. However, this is not routinely recommended in the management of tuberculosis. For adjunctive vitamin C, there was low-quality evidence showing that it may reduce the time to symptom resolution and average length of stay. However, the included studies had major limitations and further high-quality studies are needed prior to the routine use of vitamin C for severe pneumonia. Finally, for combined micronutrients, only one study provided weak evidence suggesting a small reduction in resolution of symptoms and length of hospital stay.

A limitation of this review is that 18 of the 25 individual studies excluded malnourished children or those with chronic disease for which micronutrient supplementation would likely be more beneficial. Furthermore, studies that did include these children did not perform subgroup analyses to explore the effect of micronutrient supplementation in this group. Malnourished children have immune dysfunction that increases their susceptibility to common infections [48], and conversely, children with HIV are more likely to be malnourished [49]. However, micronutrient supplementation (with zinc and a multivitamin syrup included) is already recommended as standard management for children with severe acute malnutrition by WHO, and these children should already be receiving these adjunctive treatments [4]. However, the dose of vitamin D that showed benefit in the studies in this review is much larger than the amount in multivitamins and in ready-to-use-therapeutic foods (RUTF) [50]. Similarly, the amount of vitamin C in RUTF is also less than the amount used in one study [41] (50 mg per 100 g of RUTF, compared to 200 mg OD) [50]. Future research should, among else, examine the effect of increased doses of nutritional supplements in malnourished children and evaluate children with severe nutritional deficiency at baseline or other forms of malnutrition such as stunting, where children may not receive vitamin or micronutrient supplements.

Another limitation of this review is that, despite the high mortality rates reported in children with severe pneumonia, only three studies reported mortality as a clinical outcome. Also, few of the original studies described the bacterial pathogen and it was not possible to study the relationship between the pneumonia aetiology and response to adjunct therapies.

Our review did not identify any studies from LMICs on the addition of macrolides, oral steroids, or neuraminidase inhibitors to standard antibiotic treatment in children presenting with severe pneumonia. Most of the studies of neuraminidase inhibitors excluded during full-text review studied the role of oseltamivir in preventing pneumonia in children with influenza [51,52] or did not study children with severe pneumonia [53]. A Cochrane review found that neuraminidase inhibitors such as oseltamivir (Tamiflu) and zanamivir did not significantly reduce the risk of pneumonia in children with confirmed or suspected exposure to influenza (Pooled RR = 1.06, 95% CI = 0.62, 1.83) and the benefit of treatment in those with severe pneumonia is unknown [54]. Additionally, the cost of neuraminidase inhibitors may still be prohibitive for some LMICs, with zanamivir costing US$44, and oseltamivir US$44 per dose [55,56]. A Cochrane review on the role of oral corticosteroids on pneumonia outcomes included four RCTs with children, two from high-income countries and two from China; however, the studies from China only included children with *M. pneumoniae* pneumonia and were excluded from this review [57]. Three of the four RCTs found that oral steroids reduced the time to clinical cure compared to standard antibiotic treatment (Mean difference (MD) = -1.57 days, 95% CI = -2.55, -0.60). Only one study looked at length of hospital stay in bacterial pneumonia showed a significant reduction in the oral steroid group (MD = -4.70 days with steroids, 95% CI = -7.50, -1.90) [57]. Also, the benefit of additional macrolide treatment reported in studies from the US showed that children given combination β-lactam and macrolide therapy compared to β-lactam monotherapy had a reduced length of stay (adjusted RR = 0.80, 95% CI = 0.75-0.86) [58] and reduced rates of treatment failure in those aged older than 5 years (4.0% vs 12.9%, adjusted OR = 0.51, 95% CI = 0.28-0.95). However, there was no evidence of benefit in children aged five years or less [59].

## CONCLUSIONS

This review found that adjunctive therapy with zinc or vitamin A in addition to antibiotic treatment does not improve clinical outcomes in children with severe pneumonia in LMICs. High-dose vitamin D at 100 000 IU may be beneficial, although further studies are needed to determine which populations would benefit the most. However, it would be reasonable to consider a single high dose of vitamin D in children with known vitamin D deficiency given that no adverse effects were reported. There is weak evidence of clinical benefit for vitamin C and combined nutritional supplements and further high-quality studies are needed.

Future research should focus on the evaluation of adjunctive nutritional supplementation in malnourished and HIV positive children who are most likely to benefit from treatment. Also, further studies on high dose vitamin D 100 000 IU, vitamin C, and combined nutritional supplements in children with severe pneumonia are needed. Similar to some practices in high-income countries, studies of the addition of macrolide antibiotics, corticosteroids and neuraminidase inhibitors in children presenting with severe pneumonia are required, including a cost-effectiveness analysis.

## Additional material


Online Supplementary Document
