# Supplementary material for: Review of the role of additional treatments including oseltamivir, oral steroids, macrolides, and vitamin supplementation for children with severe pneumonia in low- and middle-income countries
Source: J Glob Health. 2022 Aug 22;12:10005. doi: 10.7189/jogh.12.10005 (PMC9393748; doi:10.7189/jogh.12.10005)
Supplement: Online Supplementary Document [file jogh-12-10005-s001.pdf]

Appendix S1: MEDLINE Search strategy

1. exp *Pneumonia/
2. ((respiratory adj3 infection*) or pneumonia or pneumonias or lung-inflammation* or lobitis or nonspecific-inflammatory-lung-disease* or peripneumonia or pleuropneumonia or pleuropneumonitis or pneumonic-lung* or pneumonic-pleurisy or pneumonic-pleuritis or pneumonitides or pneumonitis or pulmonal-inflammation* or pulmonary-inflammation* or pulmonic-inflammation* or bronchiolitis).tw,kf.
3. *Pneumococcal Infections/
4. *Bacteremia/
5. exp *empyema, pleural/ or exp *pleural effusion/
6. *Streptococcus pneumoniae/
7. exp *Haemophilus/
8. (bacter?emia or empyema or pleural or bronchial or bronchoalveolar or alveolar or endotracheal or tracheal).tw,kf.
9. (pneumoniae or pneumococc* or haemophilus).tw,kf,hw.
10. (4 or 5 or 8) and (6 or 7 or 9)
11. 1 or 2 or 3 or 10
12. exp *dexamethasone/ or exp *prednisolone/ or exp *adrenal cortex hormones/ or *glucocorticoids/ or exp *steroids/
13. (adelcort or antisolon or antisolone or aprednislon or aprednislone or benisolon or benisolone or berisolon or berisolone or caberdelta or capsoid or co-hydeltra or codelcortone or compresolon or cortadeltona or cortadeltone or cortalone or cortelinter or cortisolone or cotolone or dacortin or dacrotin or decaprednil or decortin-h or decortril or dehydro-cortex or dehydro-hydrocortison or dehydro-hydrocortisone or dehydrocortex or dehydrocortisol or dehydrocortisole or dehydrohydrocortison or dehydrohydrocortisone or delcortol or delta-1-17-hydroxycorticosterone-21-acetate or delta-1-hydrocortisone or delta-cortef or delta-cortril or delta-ef-cortelan or delta-f or delta-hycortol or delta-hydrocortison or delta-hydrocortisone or delta-ophticor or delta-stab or delta1-dehydrocortisol or delta1-dehydrohydrocortisone or delta1-hydrocortisone or deltacortef or deltacortenolo or deltacortil or deltacortoil or deltacortril or deltaderm or deltaglycortril or deltahycortol or deltahydrocortison or deltahydrocortisone or deltaophticor or deltasolone or deltastab or deltidrosol or deltisilone or deltisolon or deltisolone or deltolasson or deltolassone or deltosona or deltosone or dermosolon or dhasolone or di-adreson-f or di-adresone-f or diadreson-f or diadresone-f or dicortol or domucortone or encortelon or encortelone or encortolon or equisolon or fernisolone-p or glistelone or hefasolon or hostacortin-h or hydeltra or hydeltrone or hydrelta or hydrocortancyl or hydrocortidelt or hydrodeltalone or hydrodeltisone or hydroretrocortin or hydroretrocortine or inflanefran or insolone or keteocort-h or key-pred or lenisolone or leocortol or liquipred or lygal-kopftinktur-n or mediasolone or meprisolon or meprisolone or metacortalon or metacortalone or metacortandralon or metacortandralone or metacortelone or meti-derm or meticortelone or metiderm or morlone or mydrapred or neo-delta or nisolon or nisolone or nsc-9120 or nsc9120

or opredsone or panafcortelone or panafcortolone or panafort or paracortol or phlogex or pre-cortisyl or preconin or precortalon or precortancyl or precortisyl or pred-ject-50 or predacort-50 or predaject-50 or predalone-50 or predartrina or predartrine or predate or predeltilone or predisole or predisyr or predne-dome or prednecort or prednedome or prednelan or predni-coelin or predni-h-tablinen or predni-helvacort or prednicoelin or prednicort or prednicortelone or prednifor-drops or predniment or predniretard or prednis or prednisil or prednisolon or prednisolona or prednisolone or prednivet or prednorsolon or prednorsolone or predonine or predorgasolona or predorgasolone or prelon or prelone or prenilone or prenin or prenolone or preventan or prezolon or rubycort or scherisolon or scherisolona or serilone or solondo or solone or solupren or soluprene or spiricort or spolotane or sterane or sterolone or supercortisol or supercortizol or taracortelone or walesolone or wysolone).tw,kf.

14. (adrecort or adrenocot or aeroseb-dex or aflucoson or aflucosone or alfalyl or anaflogistico or anaflogistico-novobios or arcodexan or arcodexane or artrosone or azium or bidexol or calonat or cebedex or cetadexon or colofoam or corsona or cortastat or cortidex or cortidexason or cortidrona or cortidrone or cortisumman or dacortina-fuerte or dacortine-fuerte or dalalone or danasone or de-sone-la or decacortin or decadeltosona or decadeltosone or decaderm or decadion or decadran or decadron or decadronal or decadrone or decaesadril or decaject or decameth or decamethasone or decasone or decaspray or decasterolone or decdan or decilone or decofluor or dectancyl or dekacort or delladec or deltafluoren or deltafluorene or dergramin or deronil or desacort or desacortone or desadrene or desalark or desameton or desametone or desigdron or dexa-cortisyl or dexa-dabrosan or dexa-korti or dexa-scherosan or dexa-scherozon or dexa-scherozone or dexa-p or dexacen-4 or dexachel or dexacort or dexacortal or dexacorten or dexacortin or dexacortisyl or dexadabroson or dexadecadrol or dexadrol or dexagel or dexagen or dexahelvacort or dexakorti or dexalien or dexalocal or dexame or dexamecortin or dexameson or dexamesone or dexametason or dexametasone or dexameth or dexamethason or dexamethasone or dexamethazon or dexamethazone or dexamethonium or dexamonozon or dexan or dexane or dexano or dexapot or dexascheroson or dexascherozon or dexascherozone or dexason or dexasone or dexasone-s or dexinoral or dexionil or dexmethsone or dexona or dexone or dexpak or dextelan or dextenza or dextrasone or dexycu or dezone or dibasona or doxamethasone or esacortene or ex-s1 or exadion or exadione or firmalone or fluormethyl-prednisolone or fluormethylprednisolon or fluormone or fluorocort or fluorodelta or fluoromethylprednisolone or fortecortin or gammacorten or gammacortene or grosodexon or grosodexone or hemady or hexadecadiol or hexadecadrol or hexadiol or hexadrol or isnacort or isopto-dex or isopto-maxidex or isoptodex or isoptomaxidex or lokalison-f or loverine or luxazone or marvidione or maxidex or mediamethasone or megacortin or mephameson or mephamesone or metasolon or metasolone or methazon-ion or methazone-ion or methazonion or methazonione or methylfluorprednisolone or metisone-lafi or mexasone or millicorten or millicortenol or mk-125 or mk125 or mymethasone or neoforderx or neofordex or nisomethasona or novocort or nsc-34521 or nsc34521 or oftan-dexa or opticorten or opticortinol or oradexan or oradexon or oradexone or orgadrone or ozurdex or pidexon or policort or posurdex or predni-f-tablinen or predni-f or prodexona or prodexone or sanamethasone or santenson or santeson or sawasone or solurex or spoloven or sterasone or thilodexine or triamcimetil or vexamet or visumetazone or visumethazon).tw,kf.

15. (adrenal-cortex-hormone* or glucocorticoid* or glucorticoid* or glucocorticosteroid* or steroid* or corticosteroid* or corticoid*).tw,kf.

16. (pregnenedione* or pregnenolone* or hydroxypregnenolone or tetrahydrocortisol or cortodoxone or cortisone or fludrocortisone or corticosterone or triamcinolone or prednisone or paramethasone or methylprednisolone or clobetasol or beclomethasone or betamethasone or budesonide or efcortesol or hydrocortone or solu-cortef or betnelan or betnesol or deflazacort or calcort or medrone or solu-medrone or depo-medrone or kenalog or novolizer or pulmicort or symbicort or beclometasone or aerobec or asmabec or beclazone or becodisks or becotide or clenil modulite or qvar or becloforte or cortisol).tw,kf.
17. exp *administration, intravenous/ or exp *administration, oral/
18. *infusions, parenteral/ or *infusions, intravenous/
19. (intravenous* or oral* or IV or parenteral*).tw,kf.
20. (12 or 13 or 14 or 15 or 16) and (17 or 18 or 19)
21. *Antiviral Agents/
22. *Oseltamivir/
23. (gs-4071 or gs4071 or oseltamivir or tamiflu or ebilfumin or fluvir or gs-4104 or gs4104 or oseltamavir or ro-64-0796 or ro-640796 or ro640796).tw,kf.
24. *Zanamivir/
25. (dectova or gg-167 or gg167 or gr-121167 or gr121167 or gr-121167x or gr121167x or Relenza or zanamavir).tw,kf.
26. 21 or 22 or 23 or 24 or 25
27. exp *Vitamin A/
28. (a-313 or a313 or a-fil or a-mulsal or a-mulsin or a-mulsine or a-sol or a-vi-pel or a-vit* or a313 or acon or acrisina or acrisine or actifral-a or adatone or afaxin or afaxine or afilina or afiline or agiolan or alcovit-a or alfa-monovite or alfa-sir or alfaergin or alfaergine or alfamin or alfamine or alfamonovit or alfasir or alfasole or alfasterolo or alfatar or alfavena or alfavene or alfavitina or alfavitine or alfene or alphalin or alphaline or alphasterol or amulsal or amulsin or amulsine or amulvit or anatola or anatole or anavit or anti-infective-vitamin or antixerophthalmic-vitamin or aoral or apexol or apostavit or arcavit-a or asol or asteril or atav or aterapion or avibon or avimin or avimine or avipel or avipur or avit or avitabiol or avitadit or avital or avitaminum-kolin or avitan or avitana or avitane or avite or avitil or avitina or avitol or avogina or avogine or avoleum or axerodina or axerodine or axerol or axerophthol or axerophthylium or bentavit-a or bentavite-a or bio-tan or biosterol or biotan or chivibita or cytobiase or dagravit-a or davitamon-a or difvitamin-a or dohyfral-a or elageno-a or endo-a or envit-a or epiteliol or evitol-zambeletti or fletase or gadeol or gadol or halivitan or halivitane or homagenets-aoral or hydrosol or ido-a or idratene or inovitan-a or lord-factor or meditalfa or mulsal-a or multamine or nio-a-let or oleovit-a or oleovitamin-a or ophthalamin or panvita or plivi- a or prepalin or prepaline or preparato-a or primavit or quotivit or retinol or retinyl or ro-a-vit or super-a or testavol or ucemine-a or vaconex or vaflol or veroftal or vi-alpha or vi-dom-a or viadenin or vialpha or viatate or vidoma or vitadone or vitadral or vitalen-a or vitalfa or vitama or vitamin-A or vitamin-a1 or vitaplex-a or vitapur-a or vitasan-a or vitavel-a or vitpex or vogan or vogan-neu or wandervit-a or xerophthol).tw,kf.

29. exp *Ascorbic Acid/

30. (Acidum-ascorbicum or acidylina or adenex or afj-c or agrumina or allercorb or allescorb or antiscorbutic-vitamin or arcavit-c or arcavite-c or arkovital-c or ascelat or ascofar or ascomed or asconvita or ascor or ascorbate or ascorbic-acid or ascorbicap or ascorbicin or ascorbico or ascorbin or ascorbina or ascorbinic-acid or ascorbit or ascorbite or ascorbitol or ascorbivit or ascorbivite or ascorbone or ascorbutina or ascorbyl or ascorbyn or ascorcee or ascorgil or ascorin or ascormin or ascorteal or ascorval or ascorvel or ascorvit or ascorvite or ascorvitina or askorbin or austrovit-c or austrovite-c or bentavit-c or bentavite-c or c-crivit or c-ine or c-level or c-lisa or c-long or c-monovit or c-monovite or c-prana or c-rivitin or c-rivitine or c-sol or c-tamin or c-tamine or c-tonic or c-tron or c-vescent or c-vicotrat or c-vicotrate or c-vimin or c-vimine or c-vit or c-vita or c-vital or c-vitam or c-vite or c-vimin or c-will or Cantan or cantaxin or catavin-c or ce-arom or ce-limo or ce-major or ce-quin or ce-quine or ce-vi-sol or ce-vita or ce-vitan or ce-vi-sol or cebetate or cebicure or cebion or cebione or cecap or cecon or cecon-drops or cecon-solution or cecone or cecorbin or cecorbine or cecorbyl or cecorbyle or cecrisina or Cedon or Cedone or cedoxon or cedoxone or cee-500 or ceevifil or cegiolan or celaskon or celaskone or celin or celine or cenetone or cenol or cenolate or cequinyl or cereon or cergona or cescorbat or cetamican or c etamid or cetamin-knoll-australia or cetamine or cetebe or ceterapion or ceterapione or cetrinets or cevalin or cevaline or cevatine or cevex or cevibram or cevigal or cevigen or cevigol or cevilat or cevimin or cevimine or cevisol or cevit or cevita or cevitamic-acid or cevitamin or cevitaminic-acid or cevitaminum-kolin or cevitan or cevite or cevitex or cevitil or cevitol or cewin or chewcee or chivibit-c or ci-drol or ciamin or ciamina-ormo or ciergin or cifilina or cipca or cisir or citamino or cith or citoascorbina or citoxyl or citran or citravite or citritabs or citrovitamina or civigor or civitin or civitine or co-biagini or concemin or cortalex or d-ascorbic-acid or d-xyloascorbic-acid or dagrascorbin or dagravit-c or dancimin-c or davitamon-c or dayvital or delo-c or difvitamin-c or dumovit-c or dumovite-c or e-ascorbic-acid or e-xyloascorbic-acid or erftamin-c or erftamine-c or esuron or esurvit or esurvite or flavettes or godabion-c or gregovite-c or hicee or hybrin or ido-c or ikacee or inovitan-c or irocevit or irocevite or jarexin or jarexine or l-3-keto-hexuronic-acid-lactone or l-xyloascorbic-acid or lacivit or lacivite or laroscorbine or leder-c or lemascorb or levo-ascorbate or limcee or limo-ce or liqui-cee or mega-c-a-plus or myascorbin or natrascorb or novo-ascorbic or nybadol or paa-500 or parkovit-c or pharmascorbine or pharmatovit-c or pharmatovite-c or planavit-c or planavite-c or plivit-c or plivite-c or potassium-ascorbate or pro-c or proscorbin or proscorbine or redoxon or ribena or scorbacid or scorbacide or scorbettes or scorbex or scorbin-c or scorbitol or scorbumine or scottavit-c or scottavite-c or secorbate or sevalin or sigmavit-c or sigmavite-c or sodascorbate or sodium-ascorbate or sodium-l-ascorbate or sweetcee or synum-c or take-c or tanvimil-c or testascorbic or ucemine-c or upsa-c or upsa-c or vi-ci-sin or vi-dom-c or vi-c-500 or vicef or vicelat or vicetrin or vici-monico or viciman or vicin or vicitina or vicon or viforcit or viforcite or viscorin or viscorine or vita-cedol-orange or vitac or vitace or vitacee or vitaci or vitacimin or vitacimine or vitacin or vitacine or vitamin-C or vitaplex-c or vitapric or vitapur-c or vitasan-c or vitascorbin or vitascorbine or vitascorbol or vitascorbol-500 or vitelix-c or vitocee or vorange or wandervit-c or wandervite-c or witamina-c or xitix or xon-ce or xyloascorbic-acid).tw,kf.

31. exp *Vitamin D/

32. (Cholecalciferol* or hydroxycholecalciferol* or Calcifediol* or Dihydroxycholecalciferol* or Ergocalciferol* or Dihydrotachysterol* or 25-Hydroxyvitamin-D* or Colecalciferol or dihydrotachysterol or lunacalcipol).tw,kf.
33. exp *Zinc/
34. (64Zn or zincum or Zn or zinc).tw,kf.
35. (adjuvant-therap* or adjunctive-therap* or dietary-supplement* or vitamin-supplement*).tw,kf.
36. 27 or 28 or 29 or 30 or 31 or 32 or 33 or 34 or 35
37. exp *Erythromycin/ or exp *Macrolides/
38. (macrolide* or macrocyclic-lactone-antibiotic-agent* or macrotetrolide*).tw,kf.
39. (Aruzilina or atizor or azadose or azasite or azatril or azenil or azibiot or azimin or azithral or azithromycin or azitrocin or azitromax or Azitromicin or azitromicina or aziwok or azomyne or aztrin or azydrop or azyter or azythromycin or bazyt or cp-62933 or cp62993 or forcin or inedol or infectoazit or isv-401 isv401 kromicin or macrozit or Mezatrin or octavax or ordipha or ribotrex or sumamed or sunamed or tobyl or tromix or trozocina or ultreon or vinzam or xithrone or xz-450 or xz450 or zaret or zarom or zetamax or zeto or zibramax or zifin or zimericina or zistic or zithromax or zithrox or zitinn or zitrim or zitrobifan or zitrocin or zitromax or zmax).tw,kf.
40. (a56268 or a56268 or abbotic or Abbott-56268 or aeroxina or bactirel or baxin-filmtab or biaxin or biclar or bicrolid or binoklar or bremon or brevil-od or c-clarin or carimycin or celex or cefradine or clacin or clacine or clambiotic or clapharma or clari or claribid or claridar or clarikan or clarimac or claripen or clarith or clarithromycin or clarithromycina or clarithromycine or claritrol or claroma or clormicin or crixan or cylind or cyllind or dicupal or er-36469 or er36469 or gervaken or hecobac or heliclar or helitic or klacid or klacina or klaciped or klaribac or klaricid or klaridex or klaridia or klarin or klerimed or kofron or lagur or lekoklar or macladim or macladin or maclar or macrobiol or makcin or mavid or monoclarium or monozeclar or naxy or soriclar or te-031 or te031 or veclam or winclar or zeclar).tw,kf.
41. (abomacetin or acneryne or acnesol or akne-mycin or aknederm-ery-gel or aknemycin or anamycin or bonac-gel or c-solve-2 or cliniderm or deripil or duraerythromycin or e-mycin or e-base or e-glades or e-mycin or e-solve-2 or emgel or emu-v or emu-ve or emuvin or emycin or eriecu or erimycin-t or eriprodin or eritimix or eritrex or eritrocina or eritromicina or erixyl or ermycin or ermysin or ery-maxin or ery-b or ery-diolan or ery-maxin or ery-tab or eryacne or eryacnen or eryc or eryc-ld or eryc-sprinkles or eryc-125 or eryc-250 or erycen or erycette or erycin or erycinum or eryderm or erydermec or erydermer or eryfluid or erygel or eryhexal or erymax or erymaxin or erymed or erysafe or erystrat or erytab or eryth-mycin or erythelan or erythmycin or erythomycin or erythra-derm or erythran or erythro-200 or erythro-teva or erythro-statin or erythrogan or erythrogel or erythrogran or erythroguent or erythromid or erythromycin or erythromycine or erythromycinum or erythroteva or erytop or erytrarco or erytrociclin or etinycine or etrolate or etromycin or ilocap or ilocaps or iloticina or ilotycin or inderm-gel or labocne or latotryd or lederpax or mephamycin or oftalmolosa-cusi-eritromicina or oftamolets or pantodrin or pantomycin or pce or pharyngocin or primacine or r-p mycin or robimycin or romycin or roymicin or rp-mycin or rythocin or sans-acne or sansac or skid-ge-e or staticin or stiemycin or stimycine or t-stat or theramycin).tw,kf.

42. 37 or 38 or 39 or 40 or 41
43. developing countries/
44. (austere or (limited adj2 resource*) or (low adj2 resource*) or (transitioning adj econom*) or (third adj world) or LMIC or LMICs or (lami adj countr*) or (transitional adj countr*) or (low adj gdp) or (low adj gnp) or (low adj gross adj domestic) or (low adj gross adj national) or ((emerging or developing or (low adj income) or (middle adj income) or (low adj3 middle) or underdeveloped or under-developed or (less* adj developed) or underserved or under-served or deprived or poor*) and (countr* or nation*1 or econom* or population or world))).tw,kf.
45. exp africa/
46. americas/ or exp caribbean region/ or exp central america/ or latin america/ or mexico/ or exp south america/
47. europe/ or exp europe, eastern/ or exp transcaucasia/
48. antarctic regions/ or exp atlantic islands/ or exp indian ocean islands/ or exp pacific islands/
49. New Guinea/
50. asia/ or exp asia, central/ or asia, southeastern/ or borneo/ or cambodia/ or east timor/ or indonesia/ or laos/ or malaysia/ or mekong valley/ or myanmar/ or philippines/ or thailand/ or vietnam/ or asia, western/ or bangladesh/ or bhutan/ or india/ or middle east/ or afghanistan/ or iran/ or iraq/ or jordan/ or lebanon/ or oman/ or saudi arabia/ or syria/ or turkey/ or yemen/ or nepal/ or pakistan/ or sri lanka/ or far east/ or china/ or tibet/ or exp korea/ or mongolia/
51. (Afghanistan or Albania or Algeria or Angola or Antigua or Argentina or Armenia* or Aruba or Azerbaijan or Bahrain or Bangladesh or Barbados or Barbuda or Belarus or Byelarus* or Byelorussian or Belorussian or Belorus* or Belize or Benin or Bhutan or Bolivia or Bosnia or Botswana or Brasil or Brazil or Bulgaria or (Burkina adj Fas*) or (Upper adj Volta) or Burma or Burundi or Cambodia or Khmer or Kampuchea or Cameron* or Cameroon* or (Cape adj Verde) or (Cabo adj Verde) or (Central adj African adj Republic) or Chad or Chile or China or Colombia or Comoros or (Comoro adj Island*) or Comores or Mayotte or Congo or Kongo or (Cook adj Island*) or (Costa adj Rica) or (Cote adj D'ivoire) or Croatia or Cuba or Cyprus or Czech* or Djibouti or Dominica or Dominican or (East adj Timor) or (East adj Timur) or Ecuador or Egypt or El-Salvador or (Equatorial adj Guinea) or Eritrea or Estonia or Ethiopia or Fiji or (French adj Somaliland) or Futuna or Gabon or (Gabonese adj Republic) or Gambia or Gaza or (Georgia* adj Republic) or Ghana or Grenada or Guam or Guatemala or Guinea or Guiana or Guyana or Haiti or Herzeg* or Hercegovina or Honduras or Hungary or India or Indonesia or Iran or Iraq or (Ivory adj Coast) or Jamaica or Jordan or Kazakh* or Kenya or Kiribati or Korea or Kosovo or (Kyrgyz adj Republic) or Kyrgyzstan or Kirghizia or Kirghiz or Kirgizstan or Laos or (Lao* adj2 Democratic adj Republic) or (Lao* adj PDR) or Latvia or Lebanon or Lesotho or Basutoland or Liberia or Libya or Lithuania or Macedonia or Madagascar or (Magalasy adj Republic) or Malawi or Malay* or Sabah or Sarawak or Maldives or Mali or (Marshall adj Island*) or Mauritania or Mauritius or (Agalega adj Island*) or Mexico or Micronesia or Moldov* or Mongolia or Montserrat or Montenegro or Morocco or Ifni or Mozambique or Myanma* or Namibia or Nauru or Nepal or (Netherlands adj Antilles) or (Dutch adj Antilles) or (New adj Guinea) or (New adj Caledonia) or Nicaragua or Niue or Niger or Nigeria or (Northern adj Mariana adj Island*) or Nyasaland or Oman or Pakistan or Palau or Panama or (Papua adj

New adj Guinea) or PNG or Palestine or Paraguay or Peru or Philipines or Philippines or Phillipines or Phillippines or Poland or (Puerto adj Rico) or Yemen or Romania or Roumania or Rumania or Russia* or Rwanda or Ruanda or (Saint adj Kitts) or (St adj Kitts) or Nevis or (Saint adj Vincent) or (St adj Vincent) or Grenadines or Samoa* or (Navigator adj Island*) or (Saint adj Lucia) or (St adj Lucia) or (Saint adj Helena) or (St adj Helena) or (Sao adj Tome) or (Saudi adj Arabia) or Senegal or Serbia or Seychelles or (Sierra adj Leone) or Slovenia or Slovak* or (South adj Africa) or (Solomon adj Island*) or Somalia or (Sri adj Lanka) or Ceylon or Sudan or Surinam* or Swaziland or Syria or Tajikistan or Tadzhikistan or Tadjikistan or Tadzhik or Tanzania or Thailand or Tibet or Timor-Leste or Togo or (Togolese adj Republic) or Tokelau or Tonga or Trinidad or Tobago or Tunisia or Turkey or Turkmenistan or Turkmen or Tuvalu or Uganda or Ukraine or Uruguay or Urundi or USSR or (Soviet adj Union) or "Union of Soviet Socialist Republics" or Uzbekistan or Vanuatu or (New adj Hebrides) or Venezuela or Vietnam or (Viet adj Nam) or (Wallis adj2 Futuna) or (United adj Arab adj Republic) or (West adj Bank) or (West adj Indies) or Yemen or Yugoslavia or Zaire or Zambia or Zimbabwe or Rhodesia).tw,kf.

52. (africa or americas or caribbean or (central adj America) or (latin adj America) or (south adj America) or (eastern adj Europe) or Transcaucasia or antarctic or (atlantic adj island*) or (indian adj ocean adj island*) or (pacific adj island*) or polynesia or (central adj asia) or (southeast* adj asia) or (south-east* adj asia) or borneo or mekong or (western adj asia) or (middle adj east) or (far adj east)).tw,kf.
53. 43 or 44 or 45 or 46 or 47 or 48 or 49 or 50 or 51 or 52
54. (infan* or toddler* or pre-schooler* or preschooler* or kinder or kinders or kindergarten* or kinder-aged or boy or boys or girl or girls or child or children or childhood or pediatric* or paediatric* or school-age* or schoolage* or schoolchild* or schoolgirl* or schoolboy*).af.
55. *"Severity of Illness Index"/
56. exp *Hospitalization/
57. *child, hospitalized/ or *inpatients/
58. exp *Mortality/ or *Morbidity/
59. *death/ or exp *infant death/
60. (co or mo or pc).fs.
61. *Treatment Outcome/
62. *intensive care units/ or *intensive care units, pediatric/ or *respiratory care units/ or *critical care/
63. (severe or severity or inpatient* or admission* or mortalit* or death* or died or surviv* or fatal* or prevent* or control* or hospitali#ation or hospitali#ed or refer* or morbidit* or length-of-stay or icu or picu or outcome* or ventilation or ventilator* or respiratory-support or recurren* or relapse*).tw,kf.
64. exp *respiration, artificial/
65. exp *Recurrence/
66. *survival rate/

67. 55 or 56 or 57 or 58 or 59 or 60 or 61 or 62 or 63 or 64 or 65 or 66
68. 11 and (20 or 26 or 36 or 42) and 54 and 53 and 67
69. (((Coronavirus* or corona-virus* or nCov) and ("2019" or wuhan or china or chinese or hubei)) or COV-2 or COV2 or COVID-19 or COVID19 or COVID-2019 or COVID2019 or 2019-nCoV or nCov-2019 or Coronavirus-2 or Coronavirus2 or coronavirus-disease-2019 or corona-virus-disease-2019).tw,kf.
70. Betacoronavirus/ or Coronavirus Infections/
71. limit 68 to covid-19
72. Mass Drug Administration/
73. (mass adj3 (administration* or treatment* or distribution*)).tw,kf.
74. 68 not (69 or 70 or 71 or 72 or 73)
75. limit 74 to (english language and yr="2000 -Current")

Table S1: EPHPP Quality Assessment Full Scoring of Individual Studies

Author, year	Selection bias	Study design	Confounders	Blinding	Data collection methods	Withdrawal and drop-outs	OVERALL
Acevedo-Murillo 2019	Strong	Strong	Strong	Strong	Strong	Strong	Strong
Bansal 2011	Strong	Strong	Strong	Strong	Strong	Strong	Strong
Baruah 2018	Strong	Strong	Weak	Strong	Strong	Strong	Moderate
Basnet 2012	Strong	Strong	Strong	Strong	Strong	Strong	Strong
Bose 2006	Strong	Strong	Strong	Strong	Strong	Strong	Strong
Brooks 2004	Moderate	Strong	Weak	Strong	Strong	Strong	Moderate
Choudhary 2012	Moderate	Strong	Weak	Strong	Strong	Strong	Moderate
Gupta 2016	Moderate	Strong	Strong	Strong	Strong	Strong	Strong
Hashemian 2020	Strong	Strong	Weak	Strong	Strong	Strong	Moderate
Howie 2018	Strong	Strong	Strong	Strong	Strong	Strong	Strong
Khan 2014	Moderate	Weak	Weak	Moderate	Strong	Strong	Weak
Laghari 2019	Moderate	Strong	Weak	Moderate	Strong	Weak	Weak
Mahalanabis 2006	Strong	Strong	Strong	Strong	Strong	Strong	Strong
Manaseki-Holland 2010	Strong	Strong	Strong	Strong	Strong	Strong	Strong
Manohar 2015	Moderate	Strong	Weak	Strong	Strong	Weak	Weak
Rajshekhar 2016	Moderate	Strong	Weak	Moderate	Strong	Weak	Weak
Rodriguez 2005	Moderate	Strong	Strong	Strong	Strong	Strong	Strong
Sempertegui 2014	Moderate	Strong	Strong	Strong	Strong	Strong	Strong

Shah 2012	Strong	Strong	Weak	Strong	Strong	Strong	Moderate
Srinivasan 2012	Strong	Strong	Strong	Strong	Strong	Strong	Strong
Valavi 2011	Strong	Strong	Strong	Strong	Strong	Strong	Strong
Valentiner-Branth 2010	Strong	Strong	Strong	Strong	Strong	Strong	Strong
Wadwa 2013	Moderate	Strong	Strong	Strong	Moderate	Strong	Strong
Wahed 2008	Moderate	Strong	Weak	Moderate	Strong	Weak	Weak
Yuan 2016	Moderate	Strong	Strong	Weak	Strong	Moderate	Moderate
